# Temporal trends and risk factors for readmission for infections, gastrointestinal and immobility complications after an incident hospitalisation for stroke in Scotland between 1997 and 2005

**DOI:** 10.1186/s12883-014-0257-1

**Published:** 2015-01-16

**Authors:** James Lewsey, Osaretin Ebueku, Pardeep S Jhund, Michelle Gillies, Jim WT Chalmers, Adam Redpath, Andrew Briggs, Matthew Walters, Peter Langhorne, Simon Capewell, John JV McMurray, Kate MacIntyre

**Affiliations:** Health Economics and Health Technology Assessment, Institute of Health and Wellbeing, University of Glasgow, 1 Lilybank Gardens, Glasgow, G12 8RZ UK; Public Health, Institute of Health and Wellbeing, University of Glasgow, 1 Lilybank Gardens, Glasgow, G12 8RZ UK; BHF Glasgow Cardiovascular Research Centre, Institute of Cardiovascular and Medical Sciences University of Glasgow, 126 University Place, Glasgow, G12 8TA UK; Information Services Division, NHS National Services Scotland, Gyle Square, 1 South Gyle Crescent, Edinburgh, EH12 9 EB UK; Institute of Cardiovascular and Medical Sciences, University of Glasgow, Gardiner Institute, Western Infirmary, Glasgow, G11 6NT UK; Public Health, University of Liverpool, Whelan Building, Quadrangle, Liverpool, L69 3GB UK; School of Medicine, University of Tasmania, 17 Liverpool Street, Hobart, Australia

**Keywords:** Incident stroke hospitalisation, Readmission, Cumulative incidence, Competing risks

## Abstract

**Background:**

Improvements in stroke management have led to increases in the numbers of stroke survivors over the last decade and there has been a corresponding increase of hospital readmissions after an initial stroke hospitalisation. The aim of this study was to examine the one year risk of having a readmission due to infective, gastrointestinal or immobility (IGI) complications and to identify temporal trends and any risk factors.

**Methods:**

Using a cohort of first hospitalised for stroke patients who were discharged alive, time to first event (readmission for IGI complications or death) within 1 year was analysed in a competing risks framework using cumulative incidence methods. Regression on the cumulative incidence function was used to model the risks of having an outcome using the covariates age, sex, socioeconomic status, comorbidity, discharge destination and length of hospital stay.

**Results:**

There were a total of 51,182 patients discharged alive after an incident stroke hospitalisation in Scotland between 1997–2005, and 7,747 (15.1%) were readmitted for IGI complications within a year of the discharge. Comparing incident stroke hospitalisations in 2005 with 1997, the adjusted risk of IGI readmission did not increase (HR = 1.00 95% CI (0.90, 1.11). However, there was a higher risk of IGI readmission with increasing levels of deprivation (most deprived fifth vs. least deprived fifth HR = 1.16 (1.08, 1.26).

**Conclusions:**

Approximately 15 in 100 patients discharged alive after an incident hospitalisation for stroke in Scotland between 1997 and 2005 went on to have an IGI readmission within one year. The proportion of readmissions did not change over the study period but those living in deprived areas had an increased risk.

**Electronic supplementary material:**

The online version of this article (doi:10.1186/s12883-014-0257-1) contains supplementary material, which is available to authorized users.

## Background

Stroke is a global epidemic and it has been estimated that there were 16 million incident strokes and 5.7 million stroke deaths in 2005 [1]. It is the third commonest cause of death in Scotland and the most common cause of severe physical disability in Scottish adults [2]. In the past decade innovations in stroke management has lead to an increase in the number of stroke survivors, with evidence from the UK indicating that 1 year case-fatality has declined from 41.2% in 1997 to 29.2% in 2005 [3]. Alongside this decrease in case-fatality there has been an increase in the risk of hospital readmission after stroke. In the US for the years 2003 to 2006, over half of hospitalised ischaemic stroke patients who were discharged alive were readmitted within 1 year [4], which is a notably higher percentage than those reported by individual studies included in an earlier systematic review [5].

Hospital readmission is an important outcome following stroke in epidemiological terms and is also often used as an indicator of healthcare quality for institutional comparisons. Common reasons for a hospital readmission after stroke include recurrent stroke [6-8], as well as other cardiovascular events. It has been estimated that recurrent stroke and other cardiovascular events account for approximately 23% and 17%, respectively, of readmissions within 28 days of discharge from stroke [9]. Corresponding percentages for readmissions due to recurrent stroke and other cardiovascular events at 1 year post-discharge range from 6.1–18% and 10–11.9%, respectively [10,11]. These same studies and others show that readmissions for non-cardiovascular causes are also common, with falls and fractures accounting for 4.5% and gastrointestinal disease accounting for 3.5% of readmissions within 28 days [9]. For readmissions within 1 year, it has been reported that pneumonia or respiratory illness, gastrointestinal diagnoses and infection account for 14% [10], 7% [10] and 14.1–28% [11-13], respectively.

In summary, recurrent cardiovascular events following stroke have been well studied but other commonly encountered conditions which may precipitate readmission are less well understood in this population and potentially may have a more important impact [14]. Those related to infection, gastrointestinal and immobility are particularly common. Although some studies have examined predictors of overall readmission after stroke [5,15], none have looked at predictors of readmission for non-cardiovascular causes after stroke. The aim of this study therefore was to examine the one year risk of having a readmission due to infection, gastrointestinal and immobility (IGI) complications and to identify temporal trends and any risk factors.

## Methods

The Information and Statistics Division (ISD) of the National Health Service (NHS) in Scotland collects data on all discharges from NHS hospitals using the Scottish Morbidity Record Scheme (SMR01) [16]. The data include elective and emergency admissions. Data from patient case records are used to code up to six diagnoses at the time of discharge according to the World Health Organisation Classification of Diseases (ICD 9 prior to 1996, ICD 10 post 1996). These data are routinely linked to information held by National Records of Scotland relating to all deaths.

Stroke was identified by the following ICD9 and ICD10 codes (ICD10 codes are underlined): 430 (Subarachnoid Haemorrhage), 431 (Intracerebral Haemorrhage), 433 (Occlusion and stenosis of precerebral arteries), 434 (Occlusion of cerebral arteries), 436 (Acute, but ill-defined, cerebrovascular disease), I60 (Subarachnoid Haemorrhage), I61 (Intracerebral Haemorrhage), I63 (Cerebral infarction), I64 (Stroke, not specified as haemorrhage or infarction). Incident hospitalisation for stroke was defined as a hospitalisation with a principal diagnosis of stroke with no previous hospitalisation (principal or secondary diagnosis) for cerebrovascular disease (ICD9 430–434, 436–438 and ICD10 I60-I69) in the last five years. SMR identifies stroke with an accuracy of 95% when a stroke code is recorded in the principal diagnostic position [17].

The decision to study IGI readmissions was based on the literature review and also on one study author (PL) examining the list of most commonly occurring non-cardiovascular ICD diagnoses found in readmissions to hospital following the incident stroke hospitalisation. The ICD codes used to identify IGI complications (principal diagnosis only) are shown in Additional file 1. The following comorbidities were identified from the data set: diabetes, cancer, respiratory disease, heart failure, peripheral arterial disease, atrial fibrillation, essential hypertension, renal failure, coronary heart disease, rheumatic/valvular heart disease, Parkinsonism, dementia and alcohol misuse. Comorbidities were identified using the principal and secondary diagnoses for any previous hospitalisations in the past five years and secondary diagnoses recorded in the incident stroke hospitalisation. The Scottish Index of Multiple Deprivation (SIMD) was used to measure socioeconomic status, an area measure which is based on data zones (a small geographic unit of between 500 and 1000 people). This measure is based on six domains; current income, employment, health, education, access and overcrowding [18].

All analyses were carried out on patients discharged alive after the incident stroke hospitalisation between 1997 and 2005. Time to first event (readmission for IGI complications or death) was analysed in a competing risks framework using cumulative incidence methods [19]. When there are competing events, interpretation of findings is aided by showing the results for both the outcome of interest and the competing event [20], so we show results for both outcomes. Complete one year follow-up was available for all patients. Regression on the cumulative incidence function [21] was used to model the risks of having an outcome using the covariates age, sex, socioeconomic status, comorbidity, discharge destination and length of hospital stay. All covariates relate to the initial stroke hospitalisation and all the listed covariates were part of the multivariable models. A significance level of 0.05 was used throughout and all analyses were carried out using Stata v12 (StataCorp LP, Texas, USA).

The database used in our study is not publicly available. After a successful Privacy Advisory Committee application, we obtained from ISD a full patient extract for patients with ICD 9 and ICD 10 stroke codes (ISD reference: IR2008-02322).

## Results

### Demographics of individuals with an incident hospitalisation for stroke and risk of readmission

There were a total of 51,182 patients discharged alive after an incident stroke hospitalisation in Scotland between 1997–2005, and 7,747 (15.1%) were readmitted for IGI complications within a year of the discharge (see Table 1). The risk of readmission was positively associated with increasing age. There were slightly less men than women in the cohort and their risk of readmission was lower than that for women. A higher percentage of the cohort resided in areas with the highest fifth of socioeconomic status scores than the lowest fifth of socioeconomic status scores, and the risk of readmission was positively associated with increasing deprivation. The comorbidities of renal failure, heart failure and respiratory disease were associated with the highest risks of readmission. Previous history of infection, immobility and gastrointestinal complications was common (between 16-25%) and all three were associated with an increased risk of being hospitalised for the same reasons within a year of discharge after the stroke hospitalisation. Over three-quarters of patients were discharged home and the risk of readmission for this group was similar to those who were discharged into care/nursing. Increasing length of stay was positively associated with risk of readmission.

**Table 1:** **Demographics of patients readmitted and not readmitted for infections, gastrointestinal and immobility complications within one year of incident stroke hospitalisation discharge**

	**Total**	**Not Readmitted**	**Readmitted**	**% readmitted**
	**(n = 51182)**	**(n = 43435)**	**(n = 7747)**	**(overall 15.1)**
**Age group (years):**				
**<55**	6909 (13.5)	6294 (14.5)	615 (7.9)	8.9
**55 – 64**	8238 (16.1)	7293 (16.8)	945 (12.2)	11.5
**65 – 74**	13909 (27.2)	11832 (27.2)	2077 (26.8)	14.9
**75 – 84**	15413 (30.1)	12655 (29.1)	2758 (35.6)	17.9
**85+**	6713 (13.1)	5361 (12.3)	1352 (17.5)	20.1
**Sex:**				
**Men**	24754 (48.4)	21170 (48.7)	3584 (46.3)	14.5
**Women**	26428 (51.6)	22265 (51.3)	4163 (53.7)	15.8
**Socioeconomic status:**				
**1 (least deprived)**	6805 (13.3)	5855 (13.5)	950 (12.3)	14.0
**2**	8398 (16.4)	7149 (16.5)	1249 (16.1)	14.9
**3**	10265 (20.1)	8727 (20.0)	1538 (19.8)	15.0
**4**	11797 (23.1)	9934 (22.8)	1863 (24.1)	15.8
**5 (most deprived)**	13060 (25.5)	10942 (25.2)	2118 (27.3)	16.2
**Missing**	857 (1.7)	828 (2.0)	29 (0.4)	
**Comorbidity:**				
**No comorbidity**	12724 (24.9)	11432 (26.2)	1292 (16.7)	10.2
**Diabetes**	5946 (11.6)	4862 (11.2)	1084 (14.0)	18.2
**Cancer**	3569 (7.0)	2904 (6.7)	665 (8.6)	18.6
**Respiratory disease**	4270 (8.3)	3338 (7.7)	932 (12.3)	21.8
**Heart failure**	3321 (6.5)	2570 (5.9)	751 (9.7)	22.6
**Peripheral arterial disease**	3078 (6.0)	2492 (5.7)	586 (7.6)	19.0
**Atrial fibrillation**	7429 (14.5)	6145 (14.1)	1284 (16.6)	17.3
**Essential hypertension**	15824 (30.9)	13428 (30.9)	2396 (30.9)	15.1
**Renal failure**	1481 (2.9)	1130 (2.6)	351 (4.5)	23.7
**Coronary heart disease**	10133 (19.8)	8255 (19.0)	1878 (24.2)	18.5
**Rheumatic/Valvular heart disease**	1561 (3.1)	1284 (2.9)	277 (3.6)	17.7
**Depression**	1261 (2.5)	1023 (2.4)	238 (7.1)	18.9
**Parkinsonism**	493 (1.0)	386 (1.0)	107 (1.4)	21.7
**Dementia**	1987 (3.9)	1586 (3.7)	401 (5.2)	20.2
**Alcohol misuse**	2562 (5.0)	2021 (4.7)	541 (7.0)	21.2
**Infections**	8609 (16.8)	6675 (15.4)	1934 (25.0)	22.5
**Immobility**	8021 (15.7)	6312 (14.5)	1709 (22.1)	21.3
**Gastrointestinal**	12527 (24.5)	9715 (22.4)	2812 (36.3)	22.4
**Discharge destination:**				
**Home**	40129 (78.4)	34057 (78.4)	6072 (78.4)	15.1
**Care/Nursing**	10225 (20.0)	8712 (20.1)	1513 (19.5)	14.8
**Other**	183 (0.4)	152 (0.3)	31 (0.4)	16.9
**Missing**	645 (1.3)	514 (1.2)	131 (1.6)	
**Length of stay (days):**				
**1 – 7**	14100 (27.6)	12519 (28.8)	1581 (20.4)	11.2
**8 – 14**	9047 (17.7)	7735 (17.8)	1312 (16.9)	14.5
**15 – 21**	5248 (10.3)	4434 (10.2)	814 (10.5)	15.5
**22 – 28**	3532 (6.9)	2911 (6.7)	621 (8.0)	17.6
**29+**	19255 (37.6)	15836 (36.5)	3419 (44.1)	17.8
**Year of discharge:**				
**1997 – 1999**	17083 (33.4)	14602 (33.6)	2481 (32.0)	14.5
**2000 – 2002**	17648 (34.5)	14954 (34.4)	2694 (34.8)	15.3
**2003 – 2005**	16451 (32.1)	13879 (32.0)	2572 (33.2)	15.6

### Cumulative incidence of readmission and death

**Figure 1:**
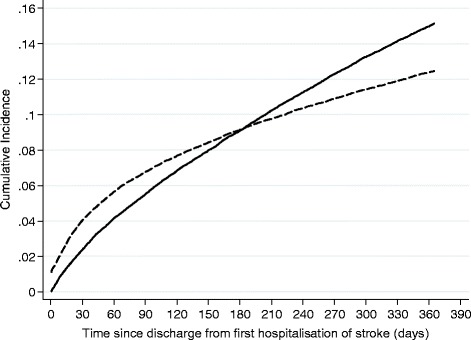
**Cumulative incidence of competing events of readmission for infections, immobility and gastrointestinal complications (solid line) and death (dashed line) within 1 year following a first hospitalization of stroke.**

Figure 1 shows the cumulative incidence of the two competing events IGI readmission and death. The risk of death is higher than the risk for readmission in the first 6 months following discharge from the stroke hospitalisation. After this point, the risk of readmission becomes higher than the risk for death and by 1 year 15.1% of patients had been readmitted and 12.5% of patients had died.

### Modelling of risk of readmission and death

After multivariable adjustment, the strength of the association between age and the risk of readmission was attenuated but remained statistically significant with those aged 85+ years having double the risk compared to those aged <55 years (see Table 2). A stronger association was observed between age and the risk of death with those aged 85+ years having over five times the risk compared to those aged <55 years. The adjusted HRs for socioeconomic status were similar for the two outcomes, showing higher risk of having an event with increasing levels of deprivation but the relative effects were much more modest than those seen for increasing age. Over the years of the study there was little evidence of a change in either the risk of readmission or risk of death. The comorbidities of diabetes, heart failure and renal failure were associated with an increased risk of readmission and risk of death. A comorbidity of atrial fibrillation and essential hypertension were both associated with a lower risk of readmission but the former was associated with a higher risk of death and the latter was associated with a lower risk of death. Patients who were discharged to long-term care/nursing homes had a 27% lower risk of readmission compared with those discharged home but had almost four times the risk of death. Multivariable adjustment greatly reduced the positive association between length of stay and the risk of death, but had less influence on the positive association between length of stay and the risk of readmission.

**Table 2:** **Unadjusted and adjusted hazard ratios from competing risks, cumulative incidence regression models for potential risk factors of readmission for infection, gastrointestinal and immobility complications and death one year after incident stroke**

	**Readmissions**		**Deaths**	
	**Unadjusted HR (95% CI)**	**Adjusted HR (95% CI)**	**Unadjusted HR (95% CI)**	**Adjusted HR (95% CI)**
**Age group (years):**				
**Age 55–64**	1.31 (1.18-1.45)	1.23 (1.11-1.36)	1.70 (1.43-2.02)	1.60 (1.35-1.90)
**Age 65–74**	1.73 (1.58-1.90)	1.58 (1.44-1.73)	3.29 (2.84-3.82)	2.52 (2.17-2.94)
**Age 75–84**	2.11 (1.94-2.31)	1.87 (1.71-2.05)	6.17 (5.35-7.13)	3.74 (3.23-4.34)
**Age 85+**	2.43 (2.21-2.68)	2.08 (1.87-2.31)	11.20 (9.67-12.95)	5.31 (4.55-6.19)
**Sex:**				
**Women**	1.09 (1.05-1.15)	1.02 (0.97-1.07)	1.31 (1.25-1.38)	0.95 (0.90-1.00)
**Socioeconomic status:**				
**2**	1.07 (0.98-1.16)	1.05 (0.96-1.14)	1.12 (1.02-1.23)	1.11 (1.01-1.22)
**3**	1.07 (0.99-1.17)	1.06 (0.97-1.15)	1.10 (1.01-1.20)	1.08 (0.99-1.19)
**4**	1.14 (1.05-1.23)	1.12 (1.03-1.21)	1.06 (0.98-1.16)	1.10 (1.01-1.20)
**5 (most deprived)**	1.17 (1.09-1.27)	1.16 (1.08-1.26)	0.98 (0.90-1.07)	1.16 (1.06-1.27)
**Year of discharge:**				
**Year 1998**	1.03 (0.93-1.14)	1.01 (0.91-1.11)	1.04 (0.94-1.15)	1.00 (0.90-1.11)
**Year 1999**	1.01 (0.92-1.12)	1.00 (0.90-1.10)	0.96 (0.86-1.07)	0.92 (0.83-1.03)
**Year 2000**	1.05 (0.95-1.16)	1.01 (0.91-1.12)	0.93 (0.84-1.03)	0.89 (0.80-0.99)
**Year 2001**	1.11 (1.01-1.22)	1.06 (0.96-1.17)	0.97 (0.87-1.07)	0.92 (0.82-1.02)
**Year 2002**	1.05 (0.95-1.16)	1.01 (0.92-1.12)	0.88 (0.79-0.98)	0.86 (0.77-0.96)
**Year 2003**	1.14 (1.03-1.26)	1.10 (0.99-1.21)	0.90 (0.81-1.00)	0.92 (0.82-1.03)
**Year 2004**	1.10 (1.00-1.21)	1.03 (0.93-1.14)	0.89 (0.79-0.99)	0.96 (0.85-1.07)
**Year 2005**	1.06 (0.96-1.17)	1.00 (0.90-1.11)	0.89 (0.80-0.99)	0.94 (0.83-1.05)
**Comorbidity:**				
**Diabetes**	1.26 (1.18-1.34)	1.17 (1.09-1.25)	1.08 (1.00-1.16)	1.11 (1.03-1.21)
**Cancer**	1.28 (1.18-1.39)	1.07 (0.98-1.16)	2.60 (2.42-2.78)	2.10 (1.95-2.27)
**Respiratory disease**	1.57 (1.46-1.68)	1.24 (1.15-1.33)	1.19 (1.10-1.30)	1.07 (0.98-1.17)
**Heart failure**	1.63 (1.51-1.76)	1.19 (1.10-1.30)	2.07 (1.92-2.23)	1.31 (1.20-1.43)
**Peripheral arterial disease**	1.30 (1.20-1.42)	1.08 (0.99-1.18)	1.34 (1.22-1.47)	1.27 (1.15-1.39)
**Atrial fibrillation**	1.18 (1.11-1.26)	0.92 (0.87-0.99)	1.63 (1.53-1.73)	1.16 (1.08-1.24)
**Essential hypertension**	0.99 (0.95-1.04)	0.91 (0.86-0.96)	0.75 (0.71-0.79)	0.79 (0.74-0.84)
**Renal failure**	1.69 (1.52-1.89)	1.23 (1.10-1.38)	2.35 (2.12-2.61)	1.67 (1.48-1.88)
**Coronary heart disease**	1.32 (1.25-1.39)	1.06 (1.00-1.12)	1.28 (1.21-1.35)	1.07 (1.00-1.15)
**Rheumatic/Valvular heart disease**	1.20 (1.06-1.35)	0.95 (0.83-1.08)	1.38 (1.22-1.57)	1.09 (0.95-1.24)
**Depression**	1.29 (1.13-1.47)	1.08 (0.94-1.23)	1.18 (1.02-1.37)	0.90 (0.77-1.06)
**Parkinsonism**	1.50 (1.23-1.81)	1.09 (0.89-1.33)	2.75 (2.34-3.23)	1.42 (1.19-1.69)
**Dementia**	1.39 (1.26-1.54)	1.06 (0.95-1.18)	2.82 (2.59-3.07)	1.26 (1.15-1.39)
**Alcohol misuse**	1.47 (1.34-1.60)	1.43 (1.30-1.57)	0.75 (0.66-0.85)	0.98 (0.85-1.12)
**Infections**	1.74 (1.65-1.83)	1.31 (1.24-1.38)	1.92 (1.81-2.03)	1.20 (1.13-1.28)
**Immobility**	1.60 (1.51-1.69)	1.23 (1.16-1.30)	1.72 (1.62-1.82)	1.01 (0.95-1.08)
**Gastrointestinal**	1.86 (1.78-1.95)	1.58 (1.51-1.66)	1.33 (1.26-1.41)	1.04 (0.99-1.11)
**Discharge destination:**				
**Care/Nursing**	0.98 (0.92-1.03)	0.73 (0.69-0.78)	5.23 (4.98-5.50)	3.75 (3.54-3.97)
**Other**	1.14 (0.80-1.64)	0.94 (0.65-1.36)	3.82 (2.83-5.15)	3.12 (2.29-4.25)
**Length of stay (days):**				
**LOS 8-14**	1.31 (1.22.1.41)	1.20 (1.11-1.29)	1.18 (1.08-1.30)	0.92 (0.83-1.01)
**LOS 15-21**	1.42 (1.30-1.55)	1.23 (1.13-1.34)	1.57 (1.42-1.74)	1.02 (0.92-1.14)
**LOS 22-28**	1.62 (1.48-1.78)	1.37 (1.24-1.51)	1.86 (1.67-2.08)	1.06 (0.94-1.18)
**LOS 29+**	1.64 (1.55-1.74)	1.41 (1.32-1.50)	2.52 (2.36-2.71)	1.10 (1.02-1.19)

Note: the reference categories for age group, sex, socioeconomic status, year of discharge, discharge destination and length of stay are <55 years, men, SIMD 1 (least deprived), 1997, home and 1 – 7 days, respectively.

**Figure 2:**
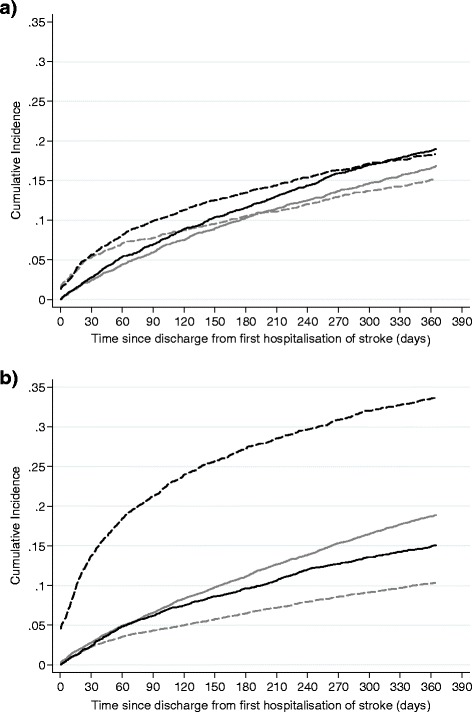
**Cumulative incidence of competing events of readmission for infections, immobility and gastrointestinal complications (solid line) and death (dashed line) within 1 year following a first hospitalization of stroke for patients 75 – 84 years old. a)** Socioeconomic status; most deprived fifth (black line) vs. least deprived fifth (grey line). **b)** Discharge destination; care/nursing (black line) vs. home (grey line).

The results in Table 2 show that the covariate effects on the two outcomes are sometimes similar but sometimes act in different directions. This is illustrated in Figure 2 where the cumulative incidences of the competing events are shown for 75 – 84 year olds for subgroups of socioeconomic status and discharge destination (a particular age group was chosen so that it did not confound the depicted associations). Figure 2a shows that the most deprived fifth of socioeconomic status has a higher level of risk of readmission and death than the least deprived fifth of socioeconomic status. In Figure 2b the much higher risk of death for those discharged to care/nursing than those discharged home can clearly be seen. For those discharged to care/nursing the risk of death was greatest in the first few months after which followed a more gradual increase out to one year. It is this strong competing risk of death that checks the risk of readmission for this group and makes it lower than the corresponding risk for those discharged home.

## Discussion

Approximately 15 in 100 patients discharged alive after an incident hospitalisation for stroke in Scotland between 1997 and 2005 were readmitted within a year because of infection, gastrointestinal and immobility complications. Contrary to expectation, the risk of IGI readmission did not change over the nine year study period. This is probably because the risk of dying before IGI readmission also stayed constant over the study period, indicating that improvements in case-fatality observed in Scotland over this time were driven by fewer patients dying in hospital and/or fewer people dying after IGI readmissions over time.

The overall percentage of readmission, 15.1%, breaks down into 4.4% for infection complications, 6.1% for gastrointestinal complications, and 4.6% for immobility complications. In the literature, the corresponding percentages for infection and gastrointestinal complications are in the range 4.6–9.0% [4,10-13] and 1.9–4.4% [4,10], respectively.

Socio-economic deprivation was shown to be a risk factor for both IGI readmission and death. It has been shown that socio-economic deprivation is a risk factor for all-cause readmissions following emergency admission for any condition, with suggested explanations being greater disease severity and poorer adherence to treatment and advice for individuals residing in areas with higher levels of deprivation [22]. To our knowledge, one other paper in the literature has reported on the association between socio-economic deprivation and risk of readmission after stroke [23]. Although not statistically significant, that paper showed higher odds of readmission for those patients with more years of education and higher incomes which is the converse of our finding. However, the earlier paper is not directly comparable as it studied all readmissions not just IGI and the follow-up period was 28 days and not 1 year.

Being discharged home was shown to be a risk factor for IGI readmission but had a reduced risk of death compared to being discharged into care/nursing, findings that are congruent with a US study [24]. Similar to other studies, we found that increasing age [12,25], increasing length of hospital stay [12], and comorbidity [25], including diabetes [26] were risk factors for having a readmission.

There are limitations to our study. First, we have used discharge codes to identify our study cohort. Internal validation studies of the Scottish Morbidity Record scheme have shown the data to identify stroke with an accuracy of 95% when recorded in a principal diagnostic position. However, the accuracy of coding is likely to vary between hospitals. Second, our risk adjustments may be inadequate because of lack of clinical detail, especially with regard the severity of the stroke. Furthermore, we could not explore whether stroke type was associated with risk of readmission because of substantial changes in the relative distribution of subarachnoid haemorrhage, intracerebral haemorrhage, cerebral infarction and ‘type not specified’ due to improvements in availability and use of imaging.

The main strengths of our study are that we studied a whole population (5.1 million people) over a 9-year period and have obtained complete one-year follow-up. Second, we considered both the previous literature and undertook an empirical approach in identifying the most common non-cardiovascular outcomes after stroke. Lastly, we employed a competing risks approach to appropriately take into account the competing risk of death.

Readmissions are an important indicator in quality improvement. Our study shows that socio-economic deprivation, as well as other demographics, need to be adjusted for before hospital performance can be compared using this indicator.

## Conclusions

In Scotland, a substantial proportion of patients hospitalised for the first time with a stroke and discharged alive are readmitted with infective, gastrointestinal or immobility complications within one year. The proportion of readmissions did not change over the nine year study period but those living in deprived areas had an increased risk.

## Additional file

Additional file 1:
**ICD classifications for infections, gastrointestinal and immobility complications.** The ICD 9 and ICD 10 codes used to identify infections, gastrointestinal and immobility complications (principal diagnosis only).

## Abbreviations

IGI: Infective, gastrointestinal or immobility complications
CI: Confidence interval
HR: Hazard ratio
ICD: International classification of diseases
ISD: Information services division
NHS: National health service
SMR: Scottish morbidity record scheme
LOS: Length of stay
