# Supplementary material for: Temporal trends and risk factors for readmission for infections, gastrointestinal and immobility complications after an incident hospitalisation for stroke in Scotland between 1997 and 2005
Source: BMC Neurol. 2015 Jan 16;15:3. doi: 10.1186/s12883-014-0257-1 (PMC4320501; doi:10.1186/s12883-014-0257-1)
Supplement: Additional file 1: — ICD classifications for infections, gastrointestinal and immobility complications. The ICD 9 and ICD 10 codes used to identify infections, gastrointestinal and immobility complications (principal diagnosis only). [file 12883_2014_257_MOESM1_ESM.pdf]

ICD classifications for infections, gastrointestinal and immobility complications

Note: ICD 10 codes with corresponding ICD 9 codes in parentheses

Infections

Intestinal infectious diseases (Other bacterial intestinal infections)

A047 (008.4)

Other bacterial diseases (Streptococcal sepsis, Other sepsis, Bacterial infection of unspecified site)

A403 (038.0), A408 (038.0), A409 (038.0), A410 (038), A4109 (038.9), A411 (038), A412 (038), A415 (038), A418 (038), A419 (038), A490 (041), A498 (041)

Infections with a predominantly sexual mode of transmission (Other predominantly sexually transmitted diseases, not elsewhere classified)

A630 (099.9)

Viral infections characterised by skin and mucous membrane lesions (Zoster [herpes zoster])

B022 (053.1), B023D (053.2), B029 (053.9)

Viral hepatitis (Chronic viral hepatitis)

B182 (070)

Other viral diseases (Viral infection of unspecified site)

B349 (079.99)

Mycoses (Candidiasis)

B378 (112.8)

Other forms of heart disease (Acute and subacute endocarditis, Endocarditis, valve unspecified)

I330 (421), I38X (424.9)

Acute upper respiratory infections (Acute pharyngitis, Acute tonsillitis, Acute upper respiratory infections of multiple and unspecified sites)

J029 (465), J039 (465), J069 (465)

Influenza and pneumonia (Influenza due to other identified influenza virus, Influenza, virus not identified, Pneumonia due to Streptococcus pneumoniae, Pneumonia due to Haemophilus influenzae, Bacterial pneumonia, not elsewhere classified, Pneumonia, organism unspecified)

J101 (487), J111 (487), J13X (481), J14X (482.2), J150 (482.0), J152 (482.4), J154 (482.3), J158 (482), J159 (482.9), J180 (485), J181 (486), J182 (514), J188 (486), J189 (486)

Other acute lower respiratory infections (Acute bronchitis, Unspecified acute lower respiratory infection)

J209 (466.0), J22X (516)

Chronic lower respiratory diseases (Bronchitis, not specified as acute or chronic)

J40X (490)

Infections of the skin and subcutaneous tissue (Cutaneous abscess, furuncle and carbuncle, Cellulitis, Other local infections of skin and subcutaneous tissue)

L021 (680), L022 (680), L023 (680), L024 (680), L030 (682), L031 (682), L039 (682), L089 (686.9)

Dermatitis and eczema (Dermatitis due to substances taken internally, Pruritus)

L270 (693.0), L299 (698.9)

Other diseases of urinary system (Cystitis, Urinary tract infection)

N300 (595), N301 (595), N302 (595), N308 (595), N390 (599)

Diseases of male genital organs (Inflammatory diseases of prostate, Orchitis and epididymitis)

N411 (601), N419 (601), N450 (604), N459 (604.0)

Inflammatory diseases of female pelvic organs (Other inflammation of vagina and vulva)

N764 (616.4)

Symptoms and signs involving the urinary system (Pain associated with micturition)

R300 (788.1)

General symptoms and signs (Fever of other and unknown origin)

R500 (780.6), R509 (780.60)

Certain early complications of trauma (Certain early complications of trauma, not elsewhere classified)

T793 (958.3)

Gastrointestinal

Intestinal infectious diseases (Other salmonella infections, Other bacterial intestinal infections, Viral and other specified intestinal infections, Other gastroenteritis and colitis of infectious and unspecified origin)

A020 (003.0), A045 (008.4), A048 (008.4), A084 (008.8), A09X (009)

Diseases of arteries, arterioles and capillaries (Aortic aneurysm and dissection)

I713 (441.3)

Diseases of veins, lymphatic vessels and lymph nodes, not elsewhere classified (Oesophageal varices)

I850 (456.1), I859 (456.1)

Diseases of oral cavity, salivary glands and jaws (Diseases of tongue)

K148 (529.8)

Diseases of oesophagus, stomach and duodenum (Oesophagitis, Gastro-oesophageal reflux disease, Other diseases of oesophagus, Gastric ulcer, Duodenal ulcer, Peptic ulcer, site unspecified, Gastritis and duodenitis, Functional dyspepsia, Other diseases of stomach and duodenum)

K20X (530.1), K210 (530.81), K219 (530.81), K220 (530.0), K221 (530.2), K222 (530.3), K224 (530.5), K225 (530.6), K226 (530.7), K228 (530.8), K229 (530.9), K250 (531), K251 (531), K253 (531), K254 (531), K255 (531), K257 (531), K259 (531.9), K260 (532), K261 (532), K263 (532), K264 (532), K265 (532), K267 (532), K269 (532.9), K279 (533), K290 (535.01), K291 (535), K292 (535.3), K293 (535), K294 (535), K295 (535), K296 (535.4), K297 (535.5), K298 (535.6), K299 (535), K30X (536.8), K311 (537.0), K315 (537.3), K317 (537.8), K318 (537.8), K319 (537.9)

Diseases of appendix (Acute appendicitis)

K350 (540), K351 (540), K359 (540)

Hernia (Inguinal hernia, Femoral hernia, Umbilical hernia, Ventral hernia, Diaphragmatic hernia, Other abdominal hernia, Unspecified abdominal hernia)

K402 (550.9), K403 (550.1), K409 (550.9), K413 (552.00), K419 (553.00), K420 (552.1), K429 (553.1), K430 (552.2), K431 (551.2), K439 (553.2), K449 (553.3), K450 (552), K460 (552.9)

Noninfective enteritis and colitis (Crohn disease [regional enteritis], Ulcerative colitis, Other noninfective gastroenteritis and colitis)

K500 (555.0), K501 (555.1), K509 (555), K512 (556.2), K519 (556.9), K520 (558.1), K522 (558.3), K528 (558.4), K529 (558)

Other diseases of intestines (Vascular disorders of intestine, Paralytic ileus and intestinal obstruction without hernia, Diverticular disease of intestine, Irritable bowel syndrome, Other functional intestinal

disorders, Fissure and fistula of anal and rectal regions, Abscess of anal and rectal regions, Other diseases of anus and rectum, Other diseases of intestine)

K550 (557.0), K552 (569.84), K558 (569.89), K559 (557.9), K560 (560.1), K562 (560.2), K563 (560.31), K564 (560.39), K565 (568.0), K566 (560.9), K570 (562.0), K571 (562.0), K572 (562), K573 (562.1), K578 (562), K579 (562), K580 (564.1), K589 (564.1), K590 (564.0), K591 (564.5), K602 (565.0), K603 (565.1), K610 (566), K620 (569.0), K621 (569.0), K623 (569.1), K624 (569.2), K625 (569.3), K626 (569.41), K627 (569.49), K628 (569.4), K631 (569.83), K635 (569.8), K638 (569.8), K639 (569.9)

Diseases of peritoneum (Peritonitis, Other disorders of peritoneum)

K650 (567.21), K659 (567.9), K660 (568.0)

Diseases of liver (Alcoholic liver disease, Hepatic failure, not elsewhere classified, Fibrosis and cirrhosis of liver, Other inflammatory liver diseases, Other diseases of liver)

K701 (571.1), K703 (571.2), K704 (571), K709 (571.3), K729 (573), K743 (571.6), K746 (571.5), K750 (572.0), K766 (572.3), K767 (572.4), K768 (573.8), K769 (573.9)

Disorders of gallbladder, biliary tract and pancreas (Cholelithiasis, Cholecystitis, Other diseases of gallbladder, Other diseases of biliary tract, Acute pancreatitis, Other diseases of pancreas)

K800 (574.0), K801 (574), K802 (574.2), K803 (576.1), K804 (574.0, 574.4), K805 (574.2), K810 (575.0), K811 (575.11), K819 (575.10), K822 (575.4), K828 (575.8), K830 (576.1), K831 (576.2), K838 (576), K839 (576.9), K85X (577.0), K860 (577.1), K861 (577.1), K863 (577.2), K868 (577.8)

Other diseases of the digestive system (Intestinal malabsorption, Postprocedural disorders of digestive system, not elsewhere classified, Other diseases of digestive system)

K900 (579.0), K914 (569.6), K918 (997.4), K920 (578.0), K921 (578.1), K922 (578.9), K929 (V12.70)

Urolithiasis (Unspecified renal colic)

N23 (788.0)

Other congenital malformations of the digestive system (Other congenital malformations of tongue, mouth and pharynx)

Q387 (750.27)

Symptoms and signs involving the digestive system and abdomen (Abdominal and pelvic pain, Nausea and vomiting, Heartburn, Dysphagia, Flatulence and related conditions, Faecal incontinence, Hepatomegaly and splenomegaly, not elsewhere classified, Unspecified jaundice, Ascites, Other symptoms and signs involving the digestive system and abdomen)

R100 (789.0), R101 (789.06), R102 (789), R103 (789.09), R104 (789.09), R11X (787.0), R12X (787.1), R13X (787.2), R14X (787.3), R15X (787.6), R161 (789.2), R17X (782.4), R18X (789.5), R190 (789.3), R194 (787.99), R195 (787.7), R198 (787.9)

General symptoms and signs (Symptoms and signs concerning food and fluid intake)

R633 (783.3), R638 (783.9)

Effects of foreign body entering through natural orifice (Foreign body in alimentary tract)

T172 (933), T181 (935)

Complications of surgical and medical care, not elsewhere classified (Complications of procedures, not elsewhere classified, Complications of cardiac and vascular prosthetic devices, implants and grafts, Complications of other internal prosthetic devices, implants and grafts, Complications peculiar to reattachment and amputation)

T814 (998.5), T827 (996.6), T855 (996.5), T874 (996.9)

Persons encountering health services for specific procedures and health care (Attention to artificial openings, Fitting and adjustment of other devices)

Z431 (V55.1), Z465 (V53.5, 55.2)

Immobility

Pulmonary heart disease and diseases of pulmonary circulation (Pulmonary embolism)

I269 (415.1)

Lung diseases due to external agents (Pneumonitis due to solids and liquids)

J690 (507.0), J698 (507.8)

Other disorders of the skin and subcutaneous tissue (Decubitus ulcer and pressure area)

L89X (707.0)

Disorders of bone density and structure (Unspecified osteoporosis with pathological fracture)

M809 (733.0)

Symptoms and signs involving the nervous and muscoskeletal systems (Abnormalities of gait and mobility, Other lack of coordination)

R260 (781.2), R262 (719.7), R268 (781.2), R270 (781.3), R278 (781.3)

Symptoms and signs involving speech and voice (Speech disturbances, not elsewhere classified)

R470 (438.1), R471 (784.5), R478 (438.1)

Injuries to the head (Superficial injury of head, Open wound of head, Fracture of skull and facial bones, Injury of eye and orbit, Intracranial injury, Other and unspecified injuries of head)

S000 (910), S001 (921), S002 (921), S007 (910), S008 (910), S009 (910), S010 (873), S011 (870), S012 (873.2), S013 (872), S018 (873), S019 (874), S021 (801), S024 (802), S026 (802.2), S051 (921), S060 (850), S062 (851), S068 (853), S069 (854), S090 (900), S099 (959.01)

Injuries to the neck (Fracture of neck, Other and unspecified injuries of neck)

S122 (805), S199 (959.0)

Injuries to the thorax (Superficial injury of thorax, Fracture of rib(s), sternum and thoracic spine, Injury of other and unspecified intrathoracic organs, Other and unspecified injuries of thorax)

S202 (911), S208 (911), S220 (805), S223 (807), S224 (807), S270 (860.0), S272 (860.4), S299 (862)

Injuries to the abdomen, lower back, lumbar spine and pelvis (Superficial injury of abdomen, lower back and pelvis, Open wound of abdomen, lower back and pelvis, Fracture of lumbar spine and pelvis, Other and unspecified injuries of abdomen, lower back and pelvis)

S300 (922), S308 (911), S309 (911), S318 (879.6), S320 (805.4), S324 (808), S325 (808), S399 (869)

Injuries to the shoulder and upper arm (Superficial injury of shoulder and upper arm, Fracture of shoulder and upper arm, Dislocation, sprain and strain of joints and ligaments of shoulder girdle, Other and unspecified injuries of shoulder and upper arm)

S400 (912), S409 (912), S420 (810.0), S422 (812), S423 (812), S4240 (812), S429 (811), S430 (831), S499 (923.0)

Injuries to the elbow and forearm (Superficial injury of forearm, Open wound of forearm, Fracture of forearm)

S509 (913), S519 (881), S520 (813), S525 (813), S526 (813), S528 (813)

Injuries to the wrist and hand (Open wound of wrist and hand, Fracture at wrist and hand level, Other and unspecified injuries of wrist and hand)

S610 (883), S619 (881), S623 (815), S625 (816), S628 (814, 815, 816), S699 (814 – 817)

Injuries to the hip and thigh (Superficial injury of hip and thigh, Fracture of femur, Dislocation, sprain and strain of joint and ligaments of hip, Other and specified injuries of hip and thigh)

S700 (924), S708 (916), S709 (916), S720 (820), S721 (820), S722 (820), S723 (821), S724 (821), S728 (821), S729 (821), S730 (835), S799 (959.6)

Injuries to the knee and lower leg (Superficial injury of lower leg, Open wound of lower leg, Fracture of lower leg, including ankle, Dislocation, sprain and strain of joints and ligaments of knee, Other and unspecified injuries of lower leg)

S800 (916), S801 (916), S808 (916), S809 (916), S818 (891), S819 (891), S820 (823 – 824), S821 (823), S823 (823), S824 (823), S825 (824), S826 (824), S828 (827), S836 (836), S899 (959)

Injuries to the ankle and foot (Superficial injury of ankle and foot, Fracture of foot, except ankle, Dislocation, sprain and strain of joints and ligaments at ankle and foot level, Other and unspecified injuries of ankle and foot)

S909 (916 – 917), S920 (827), S923 (825), S934 (845), S999 (959)

Injuries to unspecified part of trunk, limb or body region (Other injuries of lower limb, level unspecified, Injury of unspecified body region)

T130 (916), T140 (919), T149 (959)
